# Disease-associated inosine misincorporation into RNA hinders translation

**DOI:** 10.1093/nar/gkac709

**Published:** 2022-08-18

**Authors:** Jacob H Schroader, Lindsey A Jones, Ryan Meng, Hannah K Shorrock, Jared I Richardson, Sharon M Shaughnessy, Qishan Lin, Thomas J Begley, J Andrew Berglund, Gabriele Fuchs, Mark T Handley, Kaalak Reddy

**Affiliations:** The RNA Institute, University at Albany, State University of New York, Albany, NY 12222, USA; Department of Biological Sciences, University at Albany, State University of New York, Albany, NY 12222, USA; The RNA Institute, University at Albany, State University of New York, Albany, NY 12222, USA; The RNA Institute, University at Albany, State University of New York, Albany, NY 12222, USA; The RNA Institute, University at Albany, State University of New York, Albany, NY 12222, USA; The RNA Institute, University at Albany, State University of New York, Albany, NY 12222, USA; Department of Biochemistry and Molecular Biology, Center for NeuroGenetics, University of Florida, Gainesville, FL 32611, USA; The RNA Institute, University at Albany, State University of New York, Albany, NY 12222, USA; The RNA Institute, University at Albany, State University of New York, Albany, NY 12222, USA; RNA Epitranscriptomics & Proteomics Resource, University at Albany, Albany, NY 12222, USA; The RNA Institute, University at Albany, State University of New York, Albany, NY 12222, USA; Department of Biological Sciences, University at Albany, State University of New York, Albany, NY 12222, USA; RNA Epitranscriptomics & Proteomics Resource, University at Albany, Albany, NY 12222, USA; The RNA Institute, University at Albany, State University of New York, Albany, NY 12222, USA; Department of Biological Sciences, University at Albany, State University of New York, Albany, NY 12222, USA; Department of Biochemistry and Molecular Biology, Center for NeuroGenetics, University of Florida, Gainesville, FL 32611, USA; The RNA Institute, University at Albany, State University of New York, Albany, NY 12222, USA; Department of Biological Sciences, University at Albany, State University of New York, Albany, NY 12222, USA; Faculty of Biological Sciences, University of Leeds, Leeds LS2 9JT, UK; The RNA Institute, University at Albany, State University of New York, Albany, NY 12222, USA; Department of Biological Sciences, University at Albany, State University of New York, Albany, NY 12222, USA

## Abstract

Failure to prevent accumulation of the non-canonical nucleotide inosine triphosphate (ITP) by inosine triphosphate pyrophosphatase (ITPase) during nucleotide synthesis results in misincorporation of inosine into RNA and can cause severe and fatal developmental anomalies in humans. While the biochemical activity of ITPase is well understood, the pathogenic basis of ITPase deficiency and the molecular and cellular consequences of ITP misincorporation into RNA remain cryptic. Here, we demonstrate that excess ITP in the nucleotide pool during *in vitro* transcription results in T7 polymerase-mediated inosine misincorporation in luciferase RNA. *In vitro* translation of inosine-containing luciferase RNA reduces resulting luciferase activity, which is only partly explained by reduced abundance of the luciferase protein produced. Using Oxford Nanopore Direct RNA sequencing, we reveal inosine misincorporation to be stochastic but biased largely towards misincorporation in place of guanosine, with evidence for misincorporation also in place of cytidine, adenosine and uridine. Inosine misincorporation into RNA is also detected in *Itpa*-null mouse embryonic heart tissue as an increase in relative variants compared with the wild type using Illumina RNA sequencing. By generating CRISPR/Cas9 rat H9c2 *Itpa*-null cardiomyoblast cells, we validate a translation defect in cells that accumulate inosine within endogenous RNA. Furthermore, we observe hindered cellular translation of transfected luciferase RNA containing misincorporated inosine in both wild-type and *Itpa*-null cells. We therefore conclude that inosine misincorporation into RNA perturbs translation, thus providing mechanistic insight linking ITPase deficiency, inosine accumulation and pathogenesis.

## INTRODUCTION

**Figure 1.**
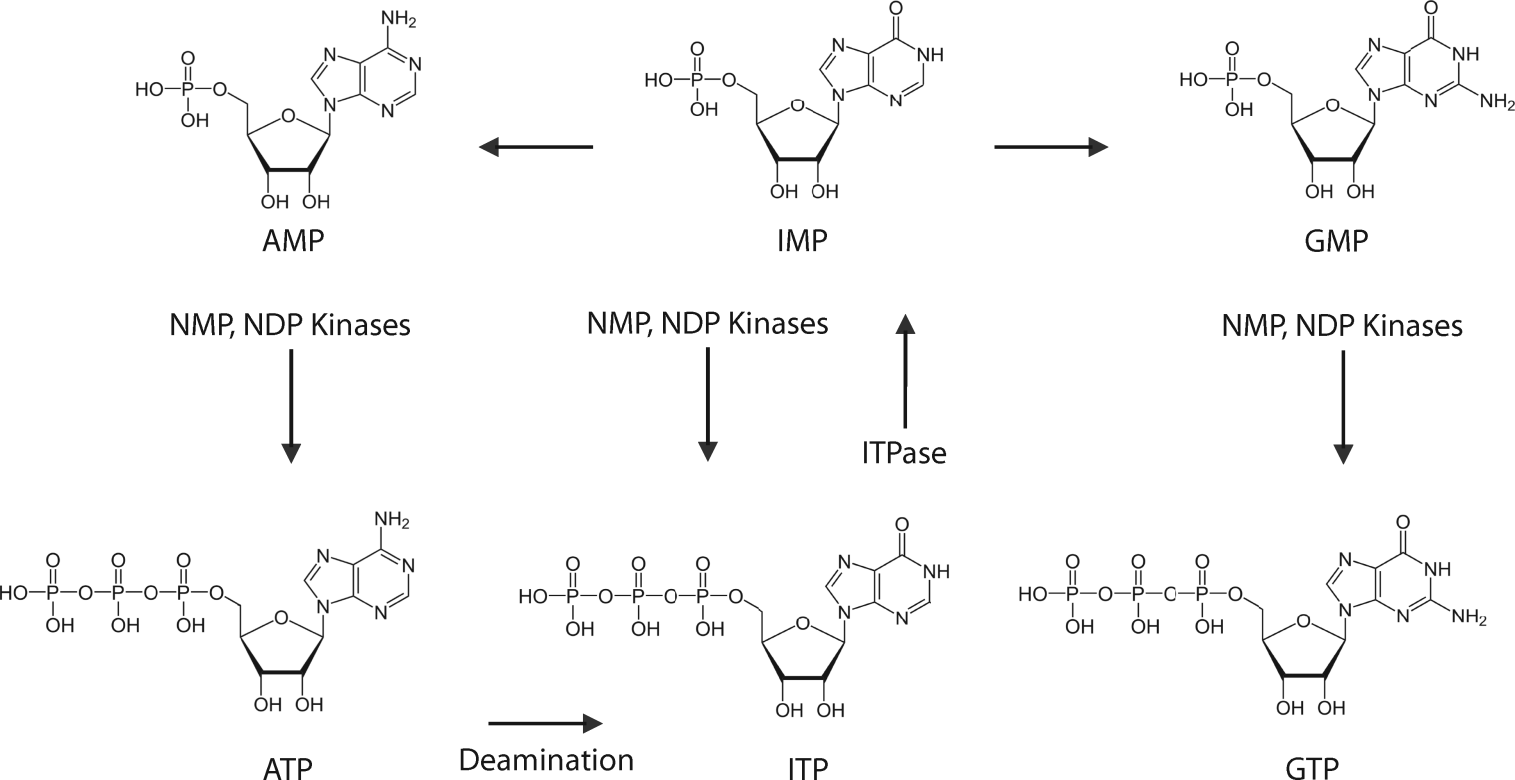
Inosine formation during nucleotide biosynthesis. IMP is a necessary precursor for the *de novo* synthesis of AMP and GMP. Either through the sequential phosphorylation of inosine mono- and diphosphate nucleotides, by nucleoside monophosphate (NMP) and nucleoside diphosphate (NDP) kinases, or through the deamination of ATP, the non-canonical nucleotide ITP may accumulate in the nucleotide pool. The aberrant accumulation of ITP can result in misincorporation into RNA. Detrimental inosine accumulation is normally restricted from the nucleotide pool through the action of inosine triphosphate pyrophosphatase (ITPase).

Inosine is most commonly known to arise in RNA as a necessary and developmentally regulated RNA A-to-I editing modification through the action of adenosine deaminase acting on RNA (ADAR) and adenosine deaminase acting on tRNA (ADAT) proteins (1–4). However, inosine may also arise in RNA aberrantly due to misincorporation of the non-canonical nucleotide inosine triphosphate (ITP) during transcription (5,6). Inosine monophosphate (IMP) is an intermediate in the generation of the nucleotides adenosine monophosphate (AMP) and guanosine monophosphate (GMP) (5,6). It is also consumed and regenerated in the ‘purine nucleotide cycle’, which is thought to regulate nucleotide pool composition and adenosine triphosphate (ATP) generation. Adventitious phosphorylation of IMP or deamination of ATP can produce the non-canonical nucleotide ITP (Figure 1).

Inosine triphosphate pyrophosphatase (ITPase) dephosphorylates ITP to prevent its accumulation in the nucleotide pool and the inappropriate incorporation of inosine into RNA during transcription (7). Variants in the *ITPA* gene encoding ITPase, leading to partial enzyme deficiency, is a relatively common trait which is clinically asymptomatic but can induce sensitivity to thiopurine therapies and modify outcomes to ribavirin therapy (8–11). More recently, we and others identified biallelic loss-of-function variants in the *ITPA* gene leading to complete loss of or severely reduced levels of ITPase, causing a fatal infantile multisystem disorder characterized by epileptic encephalopathy, microcephaly and dilated cardiomyopathy (12,13) (MIM 616647). *Itpa*-null mouse models display similar features including a neurological phenotype and dilated cardiomyopathy (14,15).

In total RNA from lymphoblastoid cells derived from an individual with complete ITPase deficiency, misincorporation of inosine was observed at a frequency of ∼1 in 5500 bases; an order of magnitude higher than that in controls (13). Moreover, examination of tissues from *Itpa*-null embryonic mice supports a correlation between the extent of inosine misincorporation and the severity of the phenotype in the affected organ. RNA from *Itpa*-null embryo heart shows inosine misincorporation at a frequency of ∼1 in 400 bases (13), while the null genotype is perinatal-lethal as a result of dilated cardiomyopathy (14). Despite extensive inosine misincorporation, microarray transcriptome analysis showed highly concordant levels of individual transcripts in *Itpa*-null and wild-type hearts (13). Although the biochemical activity of ITPase is well characterized, the molecular basis for the pathology remains cryptic and the functional consequences of inosine misincorporation into RNA are unknown.

Substitution with inosine at specific codon positions, as found within edited transcripts, was recently demonstrated to cause ribosome stalling and variable recoding effects during translation (16,17), challenging the long-held view that the translational machinery simply interprets inosine as guanosine when present in mRNA transcripts. However, the propensity of the transcription machinery to misincorporate inosine at different positions is unknown. Similarly, the effects of polymerase-mediated stochastic inosine misincorporation on translation have not been addressed. Here, we establish a highly tractable *in vitro* transcription and translation system and an *Itpa*-null rat H9c2 cardiomyoblast cell line to directly assess these phenomena. We demonstrate that excess ITP in the nucleotide pool can result in stochastic inosine misincorporation into RNA during transcription *in vitro* mostly in place of guanosine but also to a lesser extent in place of cytidine, adenosine and uridine. The resulting inosine-containing RNA poses a challenge to efficient translation *in vitro*. In cells without ITPase, inosine accumulation in RNA is accompanied by a reduced rate of translation.

## MATERIALS AND METHODS

### Plasmids

The firefly luciferase construct was purchased from Promega (L4821). To generate a parallel *Renilla* luciferase construct in the same backbone, the *Renilla* luciferase sequence (Promega E2231) was amplified by polymerase chain reaction (PCR) using the following primers: forward 5′-CATAGGATCCGCCACCATGGCTTCCAAG-3′ and reverse 5′-GCAGGGAGCTCTTACTGCTCGTTCTTC-3′, digested using BamHI and SacI, and cloned in place of firefly luciferase at the BamHI and SacI sites of Promega L4821. The Myc- and Flag-tagged firefly luciferase construct was generated by PCR amplification of Myc-firefly (GenScript) using the following primers: forward 5′-TACGAGGTACCATGAGCGAGCAGAAGCTGATCTCGG-3′ and reverse 5′-TTAACGAATTCTTAAACGGCGATCTTGCCGCCCTTC-3′, digested using KpnI and EcoRI, and cloned into those sites in pcDNA3. The C-terminal Flag tag was inserted by site-directed mutagenesis using the Q5 Site-Directed Mutagenesis Kit (NEB) with the following primers: forward 5′-GACGACGACAAGTAAGAATTCTGCAGATATCCATCACACTGG-3′ and reverse 5′-GTCCTTGTAATCAACGGCGATCTTGCCGCC-3′. Restriction digest and gel electrophoresis were performed to confirm insert sequences and sizes, and all plasmids generated were sequence verified.

### T7 *in vitro* transcription

Firefly and *Renilla* luciferase plasmids were linearized downstream of the luciferase sequence using AfeI, gel-purified and used as a template for run-off T7 phage RNA polymerase transcription (NEB HiScribe™ Kit E2050S) according to the manufacturer's instructions. Briefly, 100 ng of template was transcribed in a 20 μl volume in the presence of 10 mM of each canonical nucleotide. ITP (Sigma-Aldrich 10879) was added to a final concentration of 0, 0.1, 1 or 10 mM. Reactions were incubated at 37°C for 1 h, then treated with DNase I for an additional 15 min. RNAs were column-purified (NEB Monarch® RNA Cleanup Kit #T2040L), eluted in nuclease-free H_2_O and stored at –80°C.

### LC-MS/MS analysis of inosine and adenosine in RNA

Measurements of the level of inosine and adenosine were performed by ultra-high performance liquid chromatography coupled with tandem mass spectrometry (UHPLC-MS/MS) using a method similar to that described previously (18–20). Briefly, 200 ng of *in vitro* transcribed RNA from above or RNA extracted from H9c2 cells below (Aurum™ Total RNA Mini Kit, Bio-Rad) was digested with Nucleoside Digestion Mix (New England BioLabs) according to the manufacturer's instructions. The digested samples were then reconstituted in 100 μl of RNase-free water, 0.01% formic acid prior to UHPLC-MS/MS analysis. The UHPLC-MS/MS analysis was accomplished on a Waters XEVO TQ-S™ (Waters Corporation, USA) triple quadruple tandem mass spectrometer equipped with an electrospray ionization (ESI) source maintained at 150°C and a capillary voltage of 1 kV. Nitrogen was used as the nebulizer gas, which was maintained at 7 bars pressure, flow rate of 1000 l/h and at a temperature of 500°C. UHPLC-MS/MS analysis was performed in ESI positive ion mode using multiple-reaction monitoring (MRM) from ion transitions previously determined for inosine (*m/z* 269 > 137) and adenosine (*m/z* 268 > 136). The transition for the internal standard [guanosine (^13^C^15^N)] was *m/z* 299 > 162. A Waters ACQUITY UPLC™ HSS T3 guard column, 2.1 × 5 mm, 1.8 μm, attached to a HSS T3 column, 2.1 × 50 mm, 1.7 μm, was used for the separation. Mobile phases included RNase-free water (18 MΩcm^–1^) containing 0.01% formic acid (Buffer A) and 50% acetonitrile (v/v) in Buffer A (Buffer B). The digested nucleotides were eluted at a flow rate of 0.4 ml/min with a gradient as follows: 0–2 min, 0–10% B; 2–3 min, 10–15% B; 3–4 min, 15–100% B; 4–4.5 min, 100% B. The total run time was 7 min. The column oven temperature was kept at 35°C and the sample injection volume was 10 μl. For adenosine measurement, a 500-fold dilution of the sample was used prior to MS analysis. Data acquisition and analysis were performed using MassLynx V4.1 and TargetLynx. Calibration curves were plotted using linear regression with a weight factor of 1/*x*.

### RNA polyadenylation

*In vitro* transcribed, purified RNA was polyadenylated using the Invitrogen™ Poly(A) Tailing Kit (AM1350) according to the manufacturer's instructions. Briefly, 3 μg of RNA was incubated in a 100 μl reaction containing *Escherichia coli* poly(A) polymerase (E-PAP), 10 mM ATP and 25 mM MnCl_2_, at 37°C, for 1 h. Polyadenylated RNA was then column-purified (NEB Monarch® RNA Cleanup Kit #T2040L), eluted in nuclease-free H_2_O and stored at –80°C. Samples were electrophoresed on a 1% agarose gel containing Invitrogen™ SYBR™ Safe Gel Stain (S33102) for 1 h at 100 V, and imaged to confirm polyadenylation.

### Direct RNA nanopore sequencing

Polyadenylated luciferase RNA was used to generate libraries according to the ONT Direct RNA Sequencing (SQK-RNA002) protocol version DRS_9080_v2_revM_14Aug2019. Briefly, a minimum of 500 ng of poly(A)-tailed RNA along with 110 nM RNA CS (calibrant strand) was ligated to RT Adapter (RTA) with T4 DNA ligase (NEB M0202S) and reverse-transcribed (Thermo-Fisher SuperScript™ III 18080093), then purified using 1.8× RNAClean XP beads (Beckman Coulter® A63987), with 70% ethanol washes, on a magnetic stand. RNA Adapter (RMX) was ligated and products were purified using 1× RNAClean XP beads, with 2× WSB wash steps. The final library was eluted (ELB) and mixed with running buffer (RRB). Freshly prepared libraries were immediately loaded onto a FLO-MIN106 (R9.4.1) flow cell and sequenced using a MinION Mk1B sequencer and MinKNOW software version 19.12.5.

### Direct RNA nanopore sequencing data analysis

MinKNOW-generated FAST5 files were base called using Guppy version 4.4.2 using Q-score filtering with a minimum score of 7. Reads were mapped to the Promega T7 luciferase plasmid reference sequence using Minimap2 version 2.17 employing parameters -ax splice -uf -k14. SAMtools version 1.7 was used to convert alignment SAM files to BAM format as well as to sort and index reads using default parameters. Pysamstats version 1.1.2 was used to generate statistics text file outputs with the variation_strand parameter. RStudio version 1.4.1103 was used to calculate the accuracies for each base for reads aligning specifically to the luciferase gene of the Promega T7 luciferase plasmid reference sequence. The R package ggplot2 version 3.3.2 was used to generate the base substitution figures.

### *In vitro* translation

*In vitro* translation of luciferase RNA was conducted using a cell-free, nuclease-treated rabbit reticulocyte lysate (RRL) system (Promega L4960). Briefly, 400 ng of RNA was incubated with 7 μl of RRL, 0.1 μl of amino acid mix without cysteine (1 mM), 0.1 μl of amino acid mix without leucine (1 mM), 1 μl of RNaseOUT™ (Thermo-Fischer 10777019) and nuclease-free H_2_O to 10 μl. Samples were incubated at 30°C for 10–60 min. For radiolabelling experiments, the reactions above were modified by using 0.2 μl of amino acid mix without methionine, and 0.4 μl of 11 mCi/ml of EasyTag™ EXPRE^35^S^35^S Protein Labeling Mix (Perkin Elmer) was added and samples were incubated for 60 min. Samples were resolved by 10% sodium dodecyl sulphate (SDS)–polyacrylamide gel electrophoresis (PAGE), dried, exposed to a phosphor-screen and scanned on a Typhoon scanner (GE Amersham). Data analysis was performed using ImageQuant software (Molecular Dynamics).

### Luciferase assay

Luciferase activity was detected using the Promega Luciferase Assay System (E1500), and luminescence was read on a Promega Glomax 96 microplate luminometer. Immediately following translation at the appropriate time point, 2.5 μl of sample was mixed with the supplied cell lysis reagent. Luminescence in 96-well plates was recorded following injection of 40 μl of luciferase assay reagent into wells containing 20 μl of lysate sample.

### Illumina RNA-seq library preparation

The protocol was carried out as previously described with minor modifications (21). RNA quality was inspected via capillary electrophoresis on a Fragment Analyzer using the RNA Analysis DNF-471 kit, ensuring RNA integrity number (RIN) values >8 (Advanced Analytical). The NEBNext Ultra II Directional RNA Library Prep Kit (Illumina) with NEBNext rRNA Depletion Kit were used to prepare RNA-seq libraries, with a total of 1 μg of input RNA for the first two replicates and 500 ng for the third replicate. The protocol was carried out following the manufacturer's instructions, with the following exceptions: 40× adaptor dilutions were used; bead incubations were performed at room temperature; 4× lower concentrations of the index primers were used; and 10 cycles of library amplification were performed. The resulting libraries were pooled in equimolar quantities, quantified using the KAPA Library Quant Kit (Illumina), inspected for quality via capillary electrophoresis on a Fragment Analyzer using the NGS Analysis DNF-474 kit (Advanced Analytical) and sequenced on the Illumina NextSeq 500 massively parallel sequencer at The Center for Functional Genomics (CFG) University at Albany for the first two replicates and on the Illumina NextSeq 2000 for the third replicate at the RNA Institute, University at Albany.

### Variant analysis from RNA-seq data

FASTQ files were aligned using STAR alignment tool version 2.7.9a with parameters set to output BAM alignment files. SAMtools version 1.13 and BCFtools version 1.13 were used to create variant call files (VCFs) using the SAMtools mpileup command with the -uf options and the BCFtools call command with -mv parameters. Variants were filtered for Q scores ≥20. Pysamstats version 1.1.2 was used to generate statistics text file outputs from BAM files with the variation_strand parameter. RStudio version 1.4.1103 was used to calculate the base substitution frequencies using the statistics text files. The base substitution frequency at any particular position is defined as the number of mismatches divided by the number of matches and mismatches (total) multiplied by 100. Specific base substitutions (e.g. C>G) were calculated as described above, but specific to a particular reference and a specified alternative base. Fold changes were obtained by dividing the base substitution frequency in the *Itpa*-null library by its counterpart wild-type library.

### Cell lines

H9c2 Rat cardiomyoblast cells were obtained from the BHF Glasgow Cardiovascular Research Centre (GCRC) (Glasgow, UK) and maintained in Dulbecco’s modified Eagle’s medium (DMEM; ThermoFisher) supplemented with 10% foetal calf serum (FCS) and 1% penicillin–streptomycin at 37°C and 5% CO_2_. CRISPR/Cas9 [clustered regularly interspaced short palindromic repeats (CRISPR)/CRISPR-associated peptide 9 (Cas9)] gene editing was carried out essentially as described in Ran *et al.*, 2013 (22). A pair of gRNA sequences, 5′-gtccggttctccctgatactc-3′ and 5′-gtgtcaggaggcagctcgac-3′, targeting exon 3 of *Itpa* (ENSRNOG00000021233), were selected using the online CRISPR design tool (http://crispr.mit.edu/). Oligonucleotide pairs incorporating these sequences (Sigma) were annealed (at 50 mM each) in 10 mM Tris pH 8, 50 mM NaCl and 1 mM EDTA by incubation at 95°C for 10 min followed by cooling to room temperature. Annealed oligonucleotides were diluted and ligated into BbsI-digested pX461 and pX462 plasmids (Addgene) using HC T4 ligase and rapid ligation buffer (Promega). Sequences of the recombinant plasmids were verified by direct sequencing. Cells were transduced using a Nucleofector II device and Amaxa Cell Line Nucleofector Kit ‘L’ (Lonza Bioscience, Castleford, UK) according to the manufacturer’s instructions. Transduced cells were selected by puromycin resistance (pX462) using 24 h puromycin treatment. Following 12 h recovery, they were selected by green fluorescent protein (GFP) fluorescence (pX461) and cloned using a FACSMelody instrument (BD, Wokingham, UK). Separately cultured wild-type cells were cloned in parallel to serve as controls. After sufficient growth, clones were analysed by PCR of the targeted exon (using primers 5′-AGACAGATCATTTCGCCCTG-3′ and 5′-CAAAGAGGTTGGGTGAGAGG-3′). In order to sequence individual gene-edited alleles, PCR products from each clone were first cloned into ZeroBlunt TOPO vector (ThermoFisher) and then subjected to colony PCR. Clones with apparent loss-of-function alleles were validated by immunoblot analysis of lysates using a rabbit anti-ITPA antibody (Millipore), with anti-β-actin (ThermoFisher) serving as a loading control.

### Immunoblotting of tagged firefly luciferase protein

H9c2 cells were washed with 1× phosphate-buffered saline (PBS) 72 h post-mRNA transfection and were lysed in 200 μl of radioimmunoprecipitation assay (RIPA) buffer (ThermoScientific) with 1× cOmplete Protease Inhibitors (Roche) for 15 min on ice. DNA was sheared by passage through a 21-gauge needle, lysates were centrifuged at 21 000 × *g* for 15 min at 4°C and the supernatant was collected. The protein lysate concentration was quantified using the Pierce BCA Protein Assay Kit (ThermoScientific), and 10 μg of soluble protein lysates were separated on a 4–12% Bis–Tris gel (BioRad) and transferred to a nitrocellulose membrane (Amersham). Membranes were blocked for 2 h at room temperature in 5% dry milk in 1× PBS containing 0.05% Tween-20 (PBST; Sigma) and probed with anti-FLAG antibody (Abcam, Cat. # ab1162, 1:2000), anti-myc antibody (Abcam, Cat. # ab9106, 1:1000), anti-Firefly Luciferase antibody (Abcam, Cat. # ab185924, 1:1000) and anti-glyceraldehyde-3-phosphate dehydrogenase (GAPDH) antibody (Abcam, Cat. # ab8245, 1:10 000) overnight at 4°C in blocking solution. Following 3 × 5 min washes in 1× PBST, membranes were incubated with species-specific horseradish peroxidase (HRP)-conjugated secondary antibodies (ThermoFisher) and IRDye-680 secondary antibodies (Li-Cor) for GAPDH in blocking solution for 1 h at room tempertaure and washed 3 × 5 min in 1× PBST. Bands were visualized by using ProSignal Pico ECL Spray (Genesee Scientific) and imaging on the ChemiDoc MP Imaging System (Bio-Rad). Quantification of protein expression was performed using Image J. All protein levels are normalized within each lane to GAPDH which served as a loading control.

### Polysome profiling

Wild-type and *Itpa*-null H9c2 cells were grown to 50% confluence on 245 mm x 245 mm dishes (Corning). Cells were treated with cycloheximide (Sigma) at 100 μg/ml for 3 min, then washed with 1× PBS, 100 μg/ml cycloheximide, trypsinised and harvested by centrifugation. Washed cell pellets were resuspended in an ice-cold lysis buffer containing 150 mM NaCl, 10 mM MgCl_2_, 1 mM dithiothreitol (DTT), 1% IGEPAL, 100 μg/ml cycloheximide, Turbo DNase 24 U/μl (Invitrogen), RNasin Plus RNase Inhibitor 90 U (Promega), cOmplete Protease Inhibitor (Roche) and 50 mM Tris–HCl, pH 8, and incubated on ice for 45 min. Insoluble material was removed by centrifugation at 17 000 x *g* for 5 min at 4°C. Cytoplasmic lysates were loaded onto 18–60% sucrose gradients and subjected to ultracentrifugation (121 355 x *g*-avg, 3.5 h, 4°C). Gradients were fractionated using a Gradient station instrument (Biocomp) equipped with a detector (Biorad) for the measurement of absorbance at 254 nm, and Gradient Profiler software. Data from three technical replicate experiments were analysed. The 40S, 60S and 80S and disome peaks were defined by the lowest abutting points. For the H9C2 cell lines, clear 80S and disome peaks were not resolvable and so these peaks were considered together. Polysome peaks were defined by a distance fixed proportionate to (1.42×) the 60S–disome distance in each replicate. Absorbance measurements in each replicate were normalized according to the lowest point in each profile (the lowest point abutting 40S and 60S peaks in all cases). Polysomes:80S and disome ratios were calculated using areas under the resulting curves (trapezoid rule). *P < 0.05, Student's *t*-test.

## RESULTS

### An *in vitro* system to evaluate disease-associated inosine misincorporation

**Figure 2.**
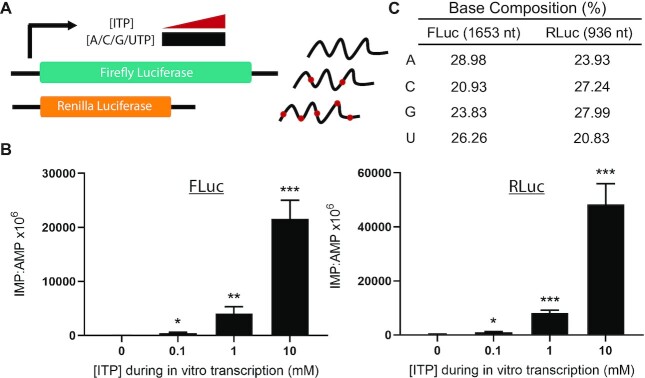
Inosine misincorporation during *in vitro* transcription. (**A**) *In vitro* transcription is initiated from linearized firefly or *Renilla* luciferase templates by the addition of T7 phage RNA polymerase in the presence of 10 mM of each canonical nucleotide. Reactions additionally contain 0.1, 1 and 10 mM ITP to simulate elevated ITP in the nucleotide pool leading to misincorporation into nascent transcripts. (**B**) Histograms showing increased inosine base content from *in vitro* transcribed firefly (FLuc) and *Renilla* (RLuc) luciferase RNA, as measured by MS. Mean ± SD, *n* = 3, unpaired two-tailed *t*-test compared with 0 mM ITP control. *P< 0.05, **P< 0.01, ***P< 0.001. (**C**) Expected length and base composition of FLuc and RLuc RNA.

Previous studies have engineered RNA to contain inosine at defined codon positions to assess the effects of inosine decoding during translation (16,17). However, the characteristics of inosine misincorporation due to elevated ITP during polymerase-mediated transcription from a DNA template have not been investigated. Furthermore, the effect of stochastic inosine misincorporation into RNA due to elevated ITP in the nucleotide pool on translation is unknown. We sought to establish a tractable *in vitro* system that enables comparisons between the level and distribution of inosine misincorporation into RNA and the production of a functional protein. Transcripts containing misincorporated inosine were generated using T7 phage RNA polymerase in an *in vitro* run-off transcription assay from linearized firefly and *Renilla* luciferase-coding plasmids. Reactions contained ITP at increasing concentrations within the nucleotide pool (0, 0.1, 1 and 10 mM), and a fixed concentration (10 mM) of each of the canonical nucleotides (Figure 2A). The resulting RNA preparations migrated as single bands following denaturing agarose gel electrophoresis (Supplementary Figure S1A). There was no evidence of truncated or abortive transcripts owing to the presence of ITP in the nucleotide pools during transcription (Supplementary Figure S1A).

### Mass spectrometry confirms inosine misincorporation during *in vitro* transcription

To detect inosine misincorporation, firefly and *Renilla* luciferase RNA preparations were digested to single nucleotides and analysed using quantitative MS (Figure 2B). Increasing levels of IMP detected in each sample corresponded to the increasing amount of ITP present in the nucleotide pool during transcription (Figure 2B). *In vitro* transcription reactions of firefly luciferase containing 0.1, 1 and 10 mM ITP resulted in detectable IMP at a level of ∼427 ± 151, 4055 ± 1285 and 21 649 ± 3369 nucleotides, respectively, per 10^6^ nucleotides of AMP, with negligible detection in the 0 mM ITP reaction (Figure 2B). These levels correspond to an inosine incorporation frequency of ∼1 in 9379, 1 in 986 and 1 in 185 bases for the 0.1, 1 and 10 mM ITP nucleotide pools, respectively. While a similar trend was observed for *Renilla* luciferase, the misincorporation rate was ∼2-fold greater with IMP detectable at a level of 1038 ± 289, 8112 ± 1039 and 48 355 ± 7666 nucleotides, respectively, per 10^6^ nucleotides of AMP in the 0.1, 1 and 10 mM reactions, with negligible detection in the 0 mM ITP reaction (Figure 2B). These levels correspond to an inosine incorporation frequency of ∼1 in 3856, 1 in 493 and 1 in 82 bases for the 0.1, 1 and 10 mM ITP nucleotide pools, respectively. The interesting disparity in inosine misincorporation between firefly and *Renilla* luciferase could potentially be attributed to the length and/or base composition differences where the *Renilla* luciferase sequence is shorter and more GC rich (Figure 2C). These incorporation rates encompass the broad range of inosine content previously observed in bulk RNA from various ITPase-deficient models including patient lymphoblastoid cell lines (∼1 in 5500 RNA bases) and mouse embryonic heart tissues (∼1 in 385 RNA bases) (13).

### Inosine misincorporation hinders translation fidelity *in vitro*

**Figure 3.**
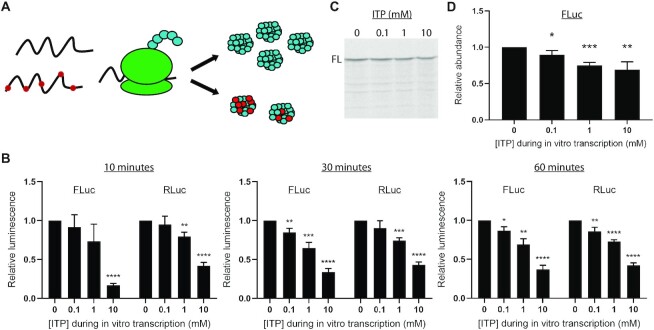
Inosine misincorporation into RNA impairs translation *in vitro*. (**A**) *In vitro* translation of luciferase RNA produces functional luciferase protein. In the presence of inosine (red dots in the RNA), ribosomes are predicted to encounter difficulties in translation which may result in a reduction in luciferase abundance and miscoding, resulting in luciferase protein with altered amino acid identities (red dots in the resulting luciferase protein). (**B**) Relative luminescence determined after *in vitro* translation of firefly (FLuc) and *Renilla* (RLuc) luciferase RNA containing misincorporated inosine compared with control RNA without inosine. Mean ± SD, *n* = 3, unpaired two-tailed *t*-test. *P< 0.05, **P< 0.01, ***P< 0.001, ****P< 0.0001. (**C**) Representative polyacrylamide gel of *in vitro* translation products in the presence of [^35^S]methionine radiolabel generated in 60 min. FL denotes full-length firefly luciferase protein. (**D**) Quantification of relative full-length firefly luciferase protein derived from three independent 60 min experiments, Mean ± SD, unpaired two-tailed *t*-test compared with 0 mM ITP control. *P< 0.05, **P< 0.01, ***P< 0.001.

To determine the functional consequences of inosine misincorporation on translation, the luciferase RNA preparations containing misincorporated inosine were evaluated using an RRL *in vitro* translation system (Promega) (Figure 3A). *In vitro* translation of inosine-containing firefly and *Renilla* luciferase RNA resulted in significant reductions in luciferase activity of the translated protein compared with protein prepared using control RNA (Figure 3B). Within 10 min of *in vitro* translation, there was a significant reduction in luminescence produced from both 10 mM ITP firefly and *Renilla* luciferase RNA (Figure 3B). Following 60 min of *in vitro* translation, there were comparable significant reductions in luminescence from all inosine-containing firefly and *Renilla* luciferase RNA preparations (Figure 3B).

Because firefly and *Renilla* RNA preparations both displayed comparable reductions in luciferase activity in the presence of inosine, we opted to focus on firefly luciferase to further probe the molecular basis of the effect of inosine misincorporation on RNA function in detail. To ensure that the reduced luciferase activity was not simply due to general base incorporation errors resulting from elevated nucleotide levels during transcription, firefly luciferase RNA preparations derived from *in vitro* transcription in the presence of 10 mM excess of each of the canonical nucleotides were evaluated (Supplementary Figure S2). MS confirmed that there was not a corresponding increase in the base incorporation rate due to 10 mM excess of any of the canonical nucleotides (Supplementary Figure S2A). Furthermore, there were no significant reductions in luciferase activity from any of these RNA preparations (Supplementary Figure S2B), supporting the observed effects to be directly due to inosine misincorporation.

To determine if the observed reduction in luciferase activity was the result of depleted or truncated luciferase protein, *in vitro* translation of inosine-containing firefly luciferase RNA was carried out in the presence of [^35^S]methionine (Figure 3C, D). There was a modest but significant relative reduction in full-length luciferase protein produced in all inosine-containing luciferase RNA templates (Figure 3C, D). There was no clear evidence of increased protein truncations resulting from the inosine-containing luciferase RNA templates (Figure 3C). These data partly explain the observed reductions in luminescence produced by the inosine-containing transcripts (Figure 3B) in terms of their reduced translation.

### Nanopore direct RNA sequencing distinguishes potential sites of inosine misincorporation

Determining the distribution of inosine misincorporation could inform on the functional outcome of translation; however, conventional polymerase-based RNA sequencing does not necessarily retain information on base modifications or non-canonical nucleotides. Efforts to detect inosine as a base modification resulting from A-to-I RNA editing at defined positions have relied on the observation that inosine preferentially pairs with cytosine during reverse transcription and so is substituted with guanosine in sequenced cDNA (23). However, inosine misincorporation at G positions would not be detectable through this approach. In contrast, Nanopore sequencing enables direct discrimination of nucleic acid bases according to the electric current generated as they are translocated through a pore protein (24–26).

To prepare samples for Nanopore sequencing, luciferase RNA generated in the presence of 0 or 10 mM ITP during *in vitro* transcription was first polyadenylated (Supplementary Figure S1B). Interestingly, the degree of polyadenylation appeared to be affected in the inosine-containing preparations, albeit modestly (Supplementary Figure S1B). Since ITP was removed from these samples, this observation suggested an effect of inosine misincorporation on polyadenylation efficiency by E-PAP (Supplementary Figure S1B).

**Figure 4.**
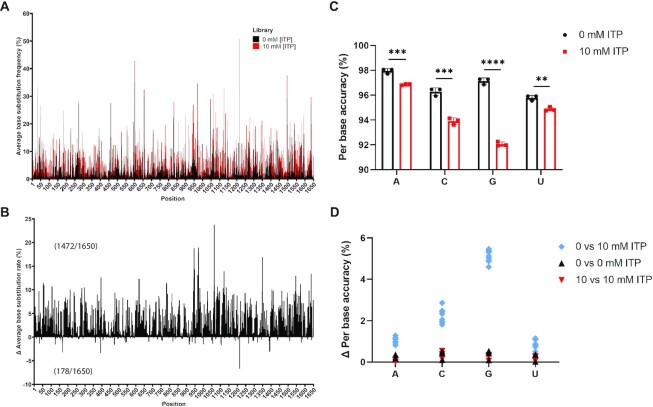
Oxford Nanopore Direct RNA sequencing detects increased base substitution errors due to stochastic inosine misincorporation. (**A**) Distribution of base substitution rates from all passed reads for each position (1–1650) of the luciferase transcript in 0 mM ITP and 10 mM ITP libraries (mean derived from *n* = 3). (**B**) Distribution of the change in base substitution rate derived by subtracting the 0 mM ITP rate from the 10 mM ITP rate for each position across the luciferase transcript (mean derived from *n* = 3). Positive values indicate a net increase in the 10 mM ITP library and negative values indicate a net increase in the 0 mM ITP libraries for any given position. The total numbers of positions with base substitution enrichment in 10 mM versus 0 mM libraries are indicated in brackets. (**C**) Average per base accuracy across the luciferase transcript derived from all passed reads. Mean ± SD, *n* = 3, unpaired two-tailed *t*-test. **P< 0.01, ***P<0.001, ****P< 0.0001. (**D**) Each pair-wise comparison of the change in (Δ) average per base accuracy for 0 mM and 10 mM ITP libraries from all three independent biological replicates.

Polyadenylated RNA was subjected to Oxford Nanopore Technologies (ONT) MinION direct RNA sequencing (SQK-RNA002). While inosine is a distinct purine nucleotide, the ONT base-calling algorithms are not currently configured to directly base call inosine, resulting in base miscalls at inosine positions which has been exploited to detect A-to-I editing sites in RNA (27). We predicted inosine misincorporation to result in increased base substitutions from base calling due to inosine-specific alterations to the expected current signals in a similar manner to previous observations for A-to-I editing (27). Therefore, we evaluated the base accuracy of aligned reads [correctly called bases/(correctly called bases + called base substitutions) × 100] as a proxy for inosine misincorporation in the 10 mM versus 0 mM luciferase RNA libraries. To determine the distribution pattern of inosine misincorporation across the luciferase gene, the average per base substitution frequency was plotted for every position of the luciferase transcript in the 0 and 10 mM libraries (Figure 4A). There was a consistent increase in base miscall frequency for the 10 mM libraries over the 0 mM libraries. While there were a few positions with peaks of base substitutions, the majority of these sites were shared between 0 and 10 mM libraries, but with an increase at those positions in the 10 mM libraries (Figure 4A). The net change in base substitution rate per position was determined, revealing a clear enrichment in the 10 mM libraries for most positions (1472/1650 sites for the 10 mM library compared with 178/1650 sites for the 0 mM library), supporting inosine misincorporation being generally stochastic (Figure 4B).

From the average base substitution frequency at each position of the luciferase RNA sequence, the average accuracy was determined for each base (Figure 4C). The average per base accuracies observed in the 0 mM libraries were between 96% and 98% with an overall hierarchy of A>G>C>U; however, in the 10 mM libraries, the accuracies dropped to between 92% and 97% and the overall hierarchy was altered to A>U>C>G (Figure 4C). These patterns were consistent across three independent biological replicates. Individual pair-wise comparisons across all three replicates of the 0 and 10 mM ITP luciferase RNA libraries revealed a clear reduction in average accuracy for each base when comparing the 0 versus 10 mM libraries, but only marginal changes when comparing 0 versus 0 mM ITP libraries and 10 versus 10 mM ITP RNA libraries (Figure 4D). The base accuracy hierarchy supported inosine misincorporation in place of guanosine to be favoured, as reflected by the greatest reduction in base accuracy (Figure 4C). Misincorporation, in descending frequency, in place of cytidine, adenosine and uridine, was nevertheless substantial. Again, to ensure misincorporation was not simply a result of altered stoichiometries due to an imbalance of available purine and pyrimidine nucleotides, Nanopore sequencing was also performed on firefly luciferase RNA generated in the presence of a 10 mM excess of either canonical CTP or GTP (Supplementary Figure S3). The base accuracies in both cases were comparable with sequencing control firefly luciferase RNA without excess nucleotides. Taken together, Nanopore direct RNA sequencing suggests inosine misincorporation is stochastic, but influenced by bases in the template DNA.

### Illumina RNA sequencing identifies elevated variants in *Itpa*-null mouse embryonic heart tissue indicative of inosine misincorporation

**Figure 5.**
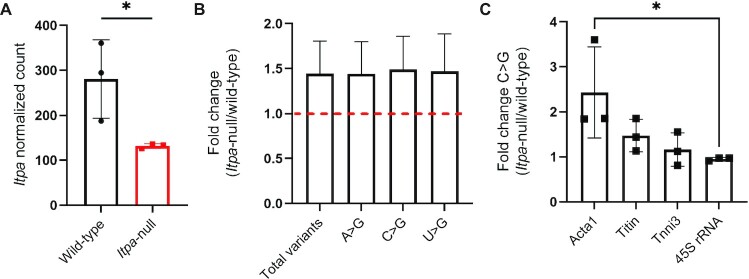
Illumina RNA sequencing identifies elevated base substitutions reflected as variants in *Itpa*-null heart tissue. (**A**) Normalized RNA counts from DESeq2 gene expression analysis reveal reduced *Itpa* normalized RNA counts in *Itpa*-null samples. Mean ± SD, *n* = 3, unpaired two-tailed *t*-test. *P< 0.05. (**B**) Total variants or variants filtered specifically by the indicated type display a modest relative increase in *Itpa*-null samples that was not statistically significant. Mean ± SD, *n* = 3. (**C**) Candidate gene analysis for C>G substitutions in *Itpa*-null samples compared with the wild type indicates elevation in select Pol II transcripts but no apparent change in Pol I-derived 45S rRNA. Individual base substitution analysis of each candidate gene was performed within a 1 kb window. The sequence window was selected by filtering for reads from the 5′ end of the gene until a continuous 1 kb 50× read depth stretch was identified. Mean ± SD, *n* = 3, one-way ANOVA with Dunnett's multiple comparisons test. *P< 0.05.

While inosine misincorporation at G positions is masked by synthesis-based RNA sequencing due to the preferential binding of inosine with cytosine, based on our *in vitro* Oxford Nanopore Direct RNA sequencing data, we hypothesized that inosine misincorporation in place of C, A and U would be detectable as an increase in C>G, A>G and U>G variants in *Itpa*-null tissue compared with the wild type. To test our hypothesis, we generated and analysed Illumina RNA-seq data from previously established *Itpa*-null mouse embryonic day 16.5 heart tissue RNA samples, where the genotype was confirmed using Sanger sequencing of *Itpa* and western blotting for ITPA protein (13). These samples were previously quantified for inosine base content using MS, revealing substantial inosine misincorporation in the *Itpa*-null samples at a level of 10 382 nucleotides IMP per 10^6^ nucleotides AMP ± 2008 SD, equating to an incorporation rate of ∼1 in 385 bases (13). *Itpa* RNA levels from the null samples were significantly reduced relative to the wild type, consistent with induction of nonsense-mediated decay resulting from the CRISPR/Cas9 genome engineering strategy (Figure 5A). Reads aligned to the mouse genome GRCm38 (mm10) were quantified for total variants, revealing a consistent but modest relative increase in the *Itpa*-null samples compared with wild-type controls across the transcriptome (Figure 5B). When variants were filtered for A>G, C>G and U>G, each type was also found to be modestly elevated in the *Itpa*-null samples (Figure 5B). These changes were not statistically significant across the transcriptome and thus we sought to evaluate a panel of candidate transcripts to determine if there were larger transcript-specific differences in base substitutions in the *Itpa*-null samples. Additionally, to determine if inosine misincorporation was independent of the RNA polymerase (Pol) involved, we attempted to quantify base substitutions from Pol I-, II- and III-derived transcripts. While there was very low read depth in our libraries from Pol III transcripts, we had substantial reads from 45S rRNA despite rRNA depletion, enabling us to quantify base substitutions as a proxy for inosine misincorporation in these RNAs. Using the candidate gene approach measuring C>G substitutions within a 1 kb window with a minimum read depth cut-off of 50× from the 5′ end of the gene, we could detect elevated C>G base substitutions at varying levels in Pol II transcripts when comparing *Itpa*-null and wild-type samples; however, there appeared to be no change in substitutions in 45S rRNA by this approach (Figure 5C). Furthermore, when we analysed A>G and U>G substitutions within the same 1 kb window for 45S rRNA, there was no observable difference, in contrast to a representative Pol II transcript (Supplementary Figure S4). Thus, RNA sequencing supports inosine misincorporation into RNA of *Itpa*-null samples *in vivo;* however, this evidence appears to be limited to a subset of Pol II transcripts.

### Inosine misincorporation hinders translation in cells

**Figure 6.**
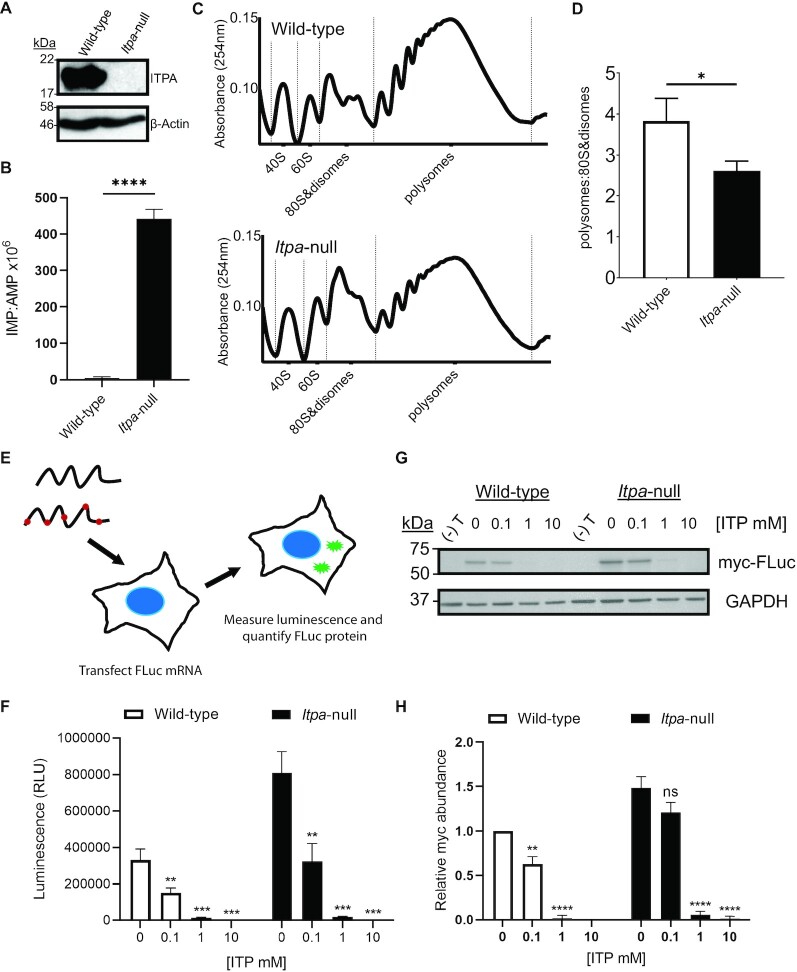
Inosine misincorporation into RNA is associated with impaired translation in cells. (**A**) Western blotting shows ITPA protein is present in wild-type H9c2 cells and absent in clonal *Itpa*-null cells generated using Cas9n and gRNAs targeting exon 3 of *Itpa*. Blotting for β-actin provides a loading control. (**B**) Histogram showing increased inosine base content of total RNA from H9c2 *Itpa*-null cells compared with the wild type measured by MS. Mean ± SD, *n* = 3, unpaired two-tailed *t*-test. ****P< 0.0001. (**C**) Representative polysome profiles of wild-type (top) and *Itpa*-null cells (bottom). Ribosomal particles were separated according to density on 18–60% sucrose gradients by ultracentrifugation. RNA absorbance at 254 nm was measured across the gradient, with peaks corresponding to ribosomal 40S and 60S subunits, 80S monosomes and disomes, and polysomes as indicated. (**D**) Active translation is reduced in *Itpa*-null cells. Polysome area under curve to 80S&disome area under curve ratios are significantly reduced in *Itpa*-null H9c2 cells compared with those in wild-type cells. Mean ± SD, unpaired two-tailed *t*-test. *P< 0.05. (**E**) Transfection of *in vitro* transcribed, capped and polyadenylated firefly luciferase RNA into wild-type and *Itpa*-null H9c2 cardiomyoblasts to evaluate effects on translation of luciferase protein by luminescence and abundance. RNA was generated in the presence of 0, 0.1, 1 and 10 mM ITP. (**F**) Luminescence in relative light units (RLU) measured 24 h after transfection of firefly luciferase RNA containing misincorporated inosine. Mean ± SD, *n* = 3, unpaired two-tailed *t*-test compared with 0 mM ITP control for each genotype. **P< 0.01, ***P<0.001. (**G**) Representative western blot using an antibody targeting the N-terminal myc tag of the firefly luciferase (FLuc) protein. (**H**) Quantification of myc-FLuc protein derived from three independent experiments using GAPDH as a protein loading control. Mean ± SD, unpaired two-tailed *t*-test compared with 0 mM ITP control for each genotype. ns = not significant, **P < 0.01, ****P< 0.0001.

In order to determine whether inosine misincorporation in RNA results in reduced translation *in vivo*, we generated an *Itpa*-null H9c2 cardiomyoblast cell line and analysed polysome profiles as a proxy for active translation. Cardiomyoblast cells were chosen because, in previous analyses of tissues, levels of inosine in RNA were most pronounced in the heart (13). CRISPR gene editing was carried out as described in Ran *et al.*, 2013 (22) using vectors for the expression of Cas9n, and a pair of gRNAs targeting exon 3 of *Itpa*. Clonal wild-type and *Itpa*-null cell lines were generated in parallel. Western blotting confirmed that ITPA is expressed in a selected wild-type clone, but was undetectable in the ITPA-null clone (Figure 6A). There was a significant enrichment of inosine in total RNA from *Itpa*-null H9c2 cells (442 ± 25 nucleotides of IMP per 10^6^ nucleotides of AMP), corresponding to an incorporation rate of ∼1 in 9037 bases of RNA (Figure 6B). This rate was similar to *in vitro* transcribed firefly luciferase RNA in the presence of 0.1 mM ITP (∼1 in 9379) which was sufficient to significantly hinder translation *in vitro* (Figure 3). Polysome profiling showed that for cells of both genotypes, peaks corresponding to 40S and 60S ribosomal subunits and polysomes were clearly distinguishable, whereas peaks corresponding to monosomes and disomes were more difficult to resolve (Figure 6C). Comparison of polysome profiles (Figure 6C, D) indicated a reduced proportion of polysomes (actively translating ribosomes) as compared with monosomes and disomes in the *Itpa*-null cells. Analysis of data from three replicate experiments showed that this reduction, expressed as a ratio of respective areas under the curves, was statistically significant (*P* < 0.05). These data are consistent with a reduced rate of global translation in the *Itpa*-null cells.

A reduced rate of translation in cells could potentially be explained by inosine misincorporation into rRNA and tRNA in addition to mRNA. Expected consequences may include increased ribosome pausing resulting from altered sequence and/or structure of the ribosome and tRNA, altered tRNA requirements or kinetics at inosine-containing codons in mRNA and increased ribosome stalling due to inosine-containing codons in mRNA. Indeed, a recent study provides direct evidence for inosine-induced ribosome stalling on mRNA (17). To aid in distinguishing the potential contribution of inosine misincorporation into rRNA and tRNA leading to a generalized defect to translation and hindered translation due to the ribosome encountering misincorporated inosine within mRNA in cells, we integrated the *in vitro* transcribed luciferase mRNA system with the *Itpa*-null and wild-type H9c2 cells. Purified, capped and polyadenylated firefly luciferase mRNA containing an N-terminal Myc tag and C-terminal Flag tag that was *in vitro* transcribed in the presence of increasing levels of ITP was transfected into wild-type and *Itpa*-null cells, and the resulting luminescence and luciferase protein abundance were evaluated (Figure 6E). In this manner, a generalized translation defect resulting from inosine misincorporation into rRNA and tRNA is expected to produce less functional luciferase protein from exogenous mRNA that does not contain inosine in *Itpa*-null cells compared with wild-type cells. Likewise, a translation defect due to inosine misincorporation into mRNA is expected to affect the resulting protein in wild-type cells that do not harbour misincorporated inosine within rRNA and tRNA. Transfection of firefly luciferase mRNA with increasing levels of misincorporated inosine resulted in a consistent significant reduction in luminescence in both wild-type and *Itpa*-null cells (Figure 6F). Surprisingly, the luminescence produced from all RNA preparations was consistently higher in the *Itpa*-null cells compared with wild-type cells, which may be the result of differential transfection efficiencies where the null cells take up more of the exogenous mRNA (Figure 6F). To ensure that the observed differences in luminescence were not attributed to variable polyadenylation in the inosine-containing mRNA preparations, a parallel experiment was conducted using the same capped firefly luciferase RNA that was *in vitro* transcribed in the presence of increasing ITP concentrations but not polyadenylated. Transfecting these non-polyadenylated firefly luciferase RNA preparations into *Itpa*-null and wild-type cells resulted in an overall reduction in luminescence as expected for non-polyadenylated RNA; however, the same pattern was retained in both cell lines compared with the polyadenylated RNA transfections, supporting the effect being independent of polyadenylation status (Supplementary Figure S5).

To evaluate protein production relative to luminescence, we carried out western blotting of the firefly luciferase protein using antibodies targeting the N-terminal Myc tag, the firefly luciferase protein directly or the C-terminal Flag tag (Figure 6G, H; Supplementary Figure S6). Full-length, tagged firefly luciferase protein of the expected size was detected using each antibody when the capped, polyadenylated firefly luciferase mRNA was transfected, and there was no evidence for truncated proteins resulting from any of the preparations. As found with luminescence, protein abundance was also higher in *Itpa*-null cells following mRNA transfection. While a similar pattern was observed using each antibody, the signal derived from the Myc-tag antibody was most robust and thus was used to quantify protein abundance (Figure 6H). Similar to findings from *in vitro* translation using RRL (Figure 3), there was a disparity in luminescence and protein abundance when comparing the 0 and 0.1 mM ITP firefly luciferase mRNA transfections in both *Itpa*-null and wild-type cells (Figure 6F–H). However, unlike the *in vitro* translation findings, there was an almost complete absence of luminescence and protein produced from the 1 and 10 mM ITP mRNA transfections in both cell lines (Figure 6F–H). Overall, our cellular studies support our conclusion that stochastic inosine misincorporation into mRNA hinders translation *in vivo*.

## DISCUSSION

Restricting the accumulation of inosine within nucleotide pools is an important biological process evidenced by the presence of *ITPA* homologues in all domains of life. Deficiency in the ITPase enzyme causes serious neurodevelopmental anomalies and dilated cardiomyopathy in mice and humans (12–15). While it is known to result in inosine misincorporation into RNA (13–15), the molecular consequences of this are unknown. We developed a luciferase T7 *in vitro* transcription and rabbit reticulocyte translation system to explore functional consequences of inosine misincorporation into RNA. Using this system, we demonstrate that titrating ITP into the NTP nucleotide pool results in a corresponding increase in misincorporated inosine within firefly and *Renilla* luciferase RNA, with a greater incorporation rate into the relatively more GC-rich *Renilla* luciferase (Figure 2). Translation of RNA containing inosine results in reduced luciferase activity which may involve a reduction in the abundance of protein produced (Figure 3). The reduction in the abundance of full-length luciferase produced was modest relative to the reduction in luciferase activity, and there was no clear evidence for truncated luciferase protein (Figure 3C), supporting the conclusion that inosine-mediated recoding also contributes to the decrease in luciferase activity observed *in vitro*. Inosine misincorporation into firefly luciferase RNA appeared to be stochastic and could occur in place of any base (Figure 4), albeit with a hierarchical base preference for G>C>A>U (Figure 4). While these observations are consistent with elevated inosine misincorporation into GC-rich transcripts, other transcript features such as expression level and length in addition to sequence composition are likely to be important determinants of inosine misincorporation. Illumina RNA sequencing supported the potential to detect inosine misincorporation *in vivo* through elevated variant detection (Figure 5). CRISPR/Cas-generated cells that are devoid of ITPase accumulate inosine in total RNA and display a translation defect (Figure 6), confirming that a translation defect is not limited to an *in vitro* setting. Furthermore, ectopically introducing firefly luciferase mRNA containing increasing inosine misincorporation into either wild-type or *Itpa*-null H9c2 cells resulted in reduced protein abundance and activity, consistent with an effect of inosine misincorporation into mRNA on translation in a cellular context. There was a striking reduction in protein abundance resulting from the 1 and 10 mM ITP luciferase mRNA in a cellular context (Figure 6G, H) that was distinct from the modest reduction in protein abundance observed from the 1 and 10 mM ITP luciferase mRNA following rabbit reticulocyte *in vitro* translation (Figure 3C, D). This observation could indicate the potential activation of cellular mRNA surveillance mechanisms in the nucleus including exosome-mediated degradation as well as cytoplasmic surveillance such as nonsense-mediated decay, non-stop decay and/or no-go decay when inosine misincorporation levels are too high (28,29). RRL, which may lack one or more of these surveillance factors, could allow for the translation of mRNA containing even very high levels of inosine misincorporation that may be efficiently targeted for degradation in cells. Future studies to identify mRNAs that contain high levels of inosine misincorporation and to systematically test the role of mRNA surveillance in degrading such mRNAs may uncover disease-relevant pathways that are perturbed in *Itpa*-null cells.

Our results provide evidence for a functional consequence of disease-associated inosine misincorporation, a phenomenon with cryptic effects on molecular and cellular function, and furnish an important clue to deciphering the enigmatic pathogenic basis of ITPase dysfunction (13). Furthermore, our study provides a starting point for future work to further dissect the effect of inosine misincorporation on translation by relating the degree of inosine misincorporation to translation activity. In this regard, it is noteworthy that as few as ∼1 inosine in every 9000 bases within the RNA population is sufficient to noticeably affect translation (Figures 3 and 6). This may be due to a combination of reduced protein production and recoding of amino acid residues, as observed in previous studies evaluating inosine at specific codon positions in the context of A-to-I editing (16,17). It is interesting that we did not observe clear evidence for protein truncation due to inosine which was previously reported to occur within codons when there were two or more inosines present but infrequently when only one inosine was present (16,17). This is likely to be a reflection of the stochastic nature of inosine distribution where even with equimolar ITP relative to the canonical nucleotides (10 mM), the misincorporation rate was up to ∼1 in 80 bases (Figure 2), probably making the occurrence of inosine clusters infrequent within a transcript.

Nanopore direct RNA sequencing and evaluation of base substitutions as a proxy for inosine misincorporation (24–27) supported a stochastic distribution (Figure 4). Furthermore, consistent with the widely held view that cells generally recognize inosine as guanosine (30), we observed a clear bias for guanosine substitutions in the inosine luciferase RNA libraries (Figure 4). Notably, however, the next most frequent substitution was cytidine (pyrimidine) rather than adenosine (a purine like inosine). While it is encouraging that Illumina RNA sequencing also identified an increase in variants in *Itpa*-null heart tissue (Figure 5), this increase was modest across the transcriptome and was not statistically significant. In contrast, when a panel of candidate transcripts were analysed, there was evidence for significantly different transcript-dependent variant levels (Figure 5C). Thus, it is of interest to determine the distribution of inosine within RNA from ITPase-deficient cells transcriptome-wide in future studies, a particularly challenging task given the stochastic nature of the resulting inosine distribution.

An open question is whether the different classes of RNA polymerases display distinct patterns of inosine misincorporation into various classes of RNA including rRNA and tRNA which, in addition to coding mRNA, could be contributing to the disruption in translation activity observed in *Itpa*-null cells (Figure 6). While Illumina RNA sequencing did not reveal an elevation in 45S rRNA base substitutions detectable from *Itpa*-null heart tissue, we cannot rule out misincorporation into rRNA or tRNA contributing to the observed cellular translation defect in *Itpa*-null cells (Figure 6). Indeed, the importance of preserving inosine function in tRNA at the wobble position (31,32) supports a crucial level of translational control imparted by the presence of inosine at particular positions in tRNA which, if deregulated through misincorporation, would be predicted to disrupt translation. Likewise, misincorporation into rRNA could have detrimental effects on translation through disrupted structural dynamics induced by the unique hydrogen bonding potential of inosine compared with guanosine as well as to the other canonical and modified nucleotides in RNA (33). Nonetheless, our RNA transfection studies support the detrimental effects of inosine misincorporation into mRNA in a cellular context (Figure 6F, G, H), but separately investigating the potential misincorporation into rRNA and tRNA remains an important future research avenue.

In the future, it will also be of interest to apply Nanopore sequencing to RNA derived from ITPase-deficient cells and tissues to better explore the transcriptomic distribution of inosine *in vivo* and to determine if there is a correlation between organ dysfunction and inosine misincorporation. In this regard, a recent study reported a neural-specific *Itpa* conditional knockout (cKO) mouse model, displaying a robust neurological condition including seizures with depolarization of the resting membrane potential and increased frequency of neuronal excitation (15). Substantial inosine misincorporation was observed in bulk RNA from the cerebral cortex, hippocampus and cerebellum in *Itpa* cKO mice compared with controls, but the mechanistic basis underlying the neural pathology was not uncovered. A challenge in elucidating the pathogenic basis of ITPase deficiency is the numerous molecular mechanisms through which ITP accumulation may disrupt cellular function. In this study, we provide evidence for a translation defect resulting from inosine misincorporation into RNA, thus providing one potential link between ITPase dysfunction and pathogenesis.

## DATA AVAILABILITY

The Oxford Nanopore Direct RNA sequencing (BioProject accession no. PRJNA743081) and Illumina RNA-seq data (BioProject accession no. PRJNA822480) have been deposited in the Sequence Read Archive (SRA) database, https://www.ncbi.nlm.nih.gov/sra.

## Supplementary Material

gkac709_Supplemental_File
