# Supplementary material for: Disease-associated inosine misincorporation into RNA hinders translation
Source: Nucleic Acids Res. 2022 Aug 18;50(16):9306–18. doi: 10.1093/nar/gkac709 (PMC9458462; doi:10.1093/nar/gkac709)
Supplement: gkac709_Supplemental_File [file gkac709_supplemental_file.pdf]

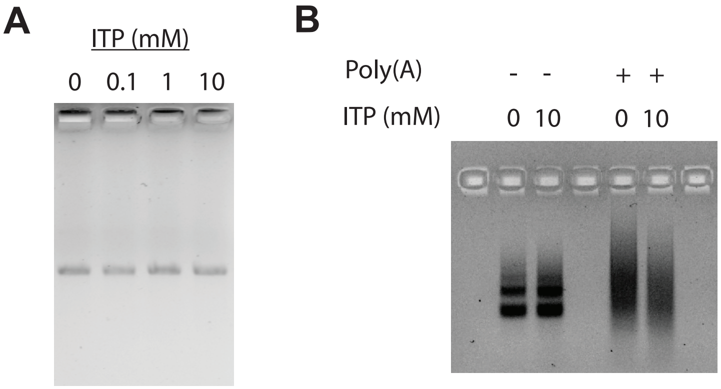

Figure S1. (A) Representative firefly luciferase RNA preperations resolved on 1% denaturing agarose gel. (B) Representative firefly luciferase RNA preperations without and with polyadenylation resolved on 1% native agarose gel.

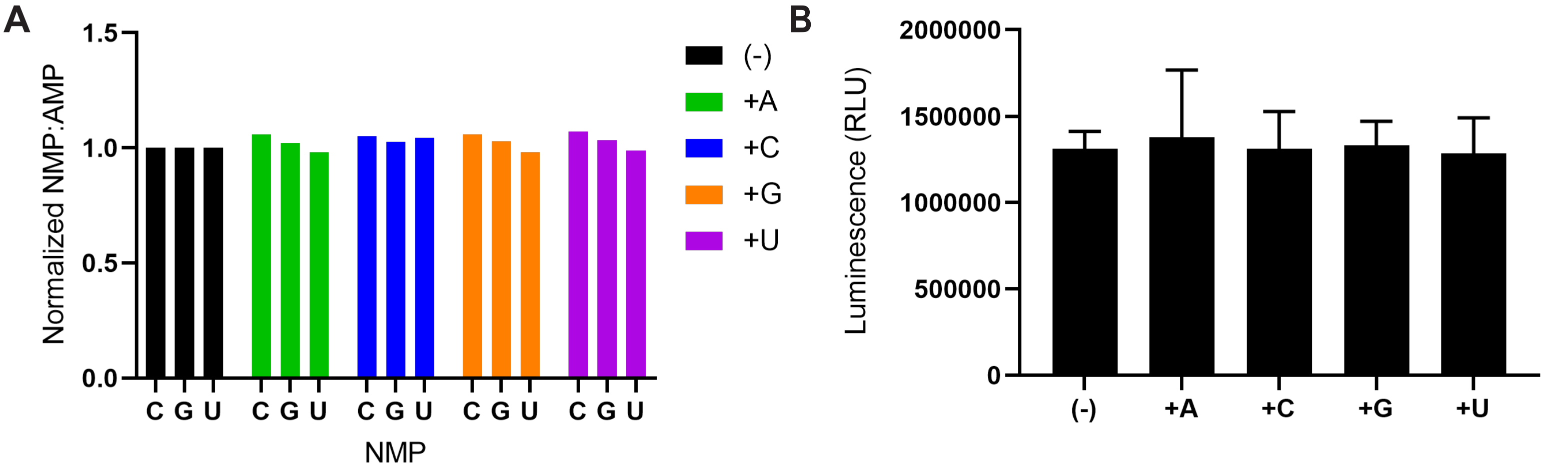

Figure S2. (A) Histogram showing base content of firefly luciferase RNA in vitro transcribed in the presence of 10 mM excess of the indicated canonical nucleotide, measured by mass spectrometry. (B) Luminescence in relative light units (RLU) measured following in vitro translation of firefly luciferase RNA that was in vitro transcribed in the presence of 10 mM excess of the indicated canonical nucleotide.

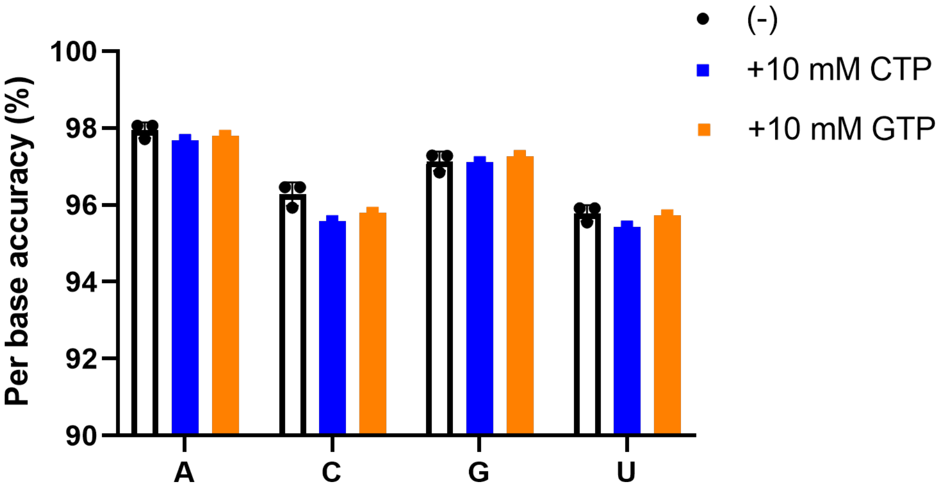

Figure S3. Nanopore direct RNA sequencing to measure average per base accuracy across the firefly luciferase transcript that was generated from in vitro transcription in the presence of 10 mM excess of the indicated canonical nucleotide.

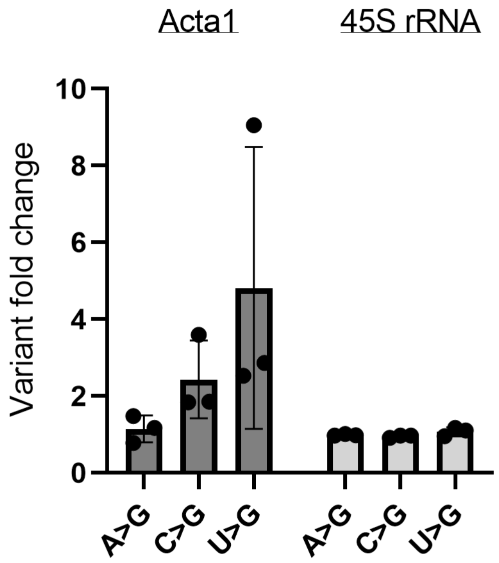

Figure S4. Base substitutions identified from RNAseq data represented as fold change by dividing percentage of variants in Itpa-null samples by wild-type. Variant analysis of each candidate gene was performed within a 1 kilobase window. The sequence window was selected by filtering for reads from the 5' end of the gene until a continuous 1 kilobase 50x read depth stretch was identified. Mean + /- SD, n=3.

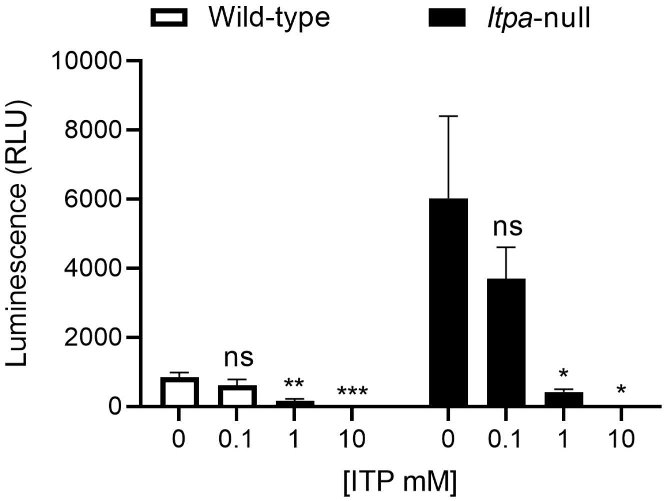

Figure S5. Luminescence in relative light units (RLU) measured 24 hours following transfection of capped, firefly luciferase RNA lacking polyadenylation into H9c2 wildtype and Itpa-null cells. The RNA was in vitro transcribed in the presence of the indicated concentration of ITP. Mean ± SD, n=3, unpaired two-tailed t-test comparing to 0mM ITP control for each genotype. *P < 0.05, **P < 0.01, ***P < 0.001.

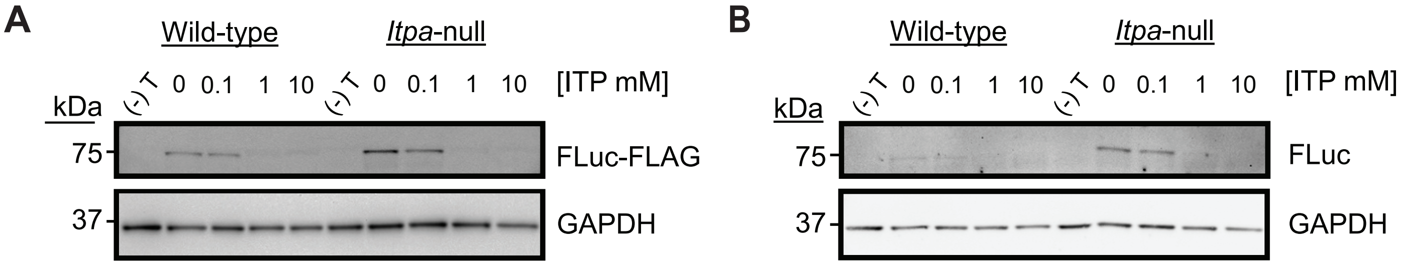

Figure S6. Detection of firefly luciferase protein produced in H9c2 wild-type and Itpa-null cells following transfection of capped, polyadenylated firefly luciferase RNA that was in vitro transcribed in the presence of the indicated concentration of ITP, (-) T indicates no transfection control. (A) Western blotting using an antibody against the FLAG tag or (B) directly against firefly luciferase protein.
